# Contact heat evoked potentials using simultaneous EEG and fMRI and their correlation with evoked pain

**DOI:** 10.1186/1471-2253-8-8

**Published:** 2008-12-17

**Authors:** Katherine Roberts, Anastasia Papadaki, Carla Gonçalves, Mary Tighe, Duncan Atherton, Ravikiran Shenoy, Donald McRobbie, Praveen Anand

**Affiliations:** 1 Imperial College Healthcare NHS Trust, London, UK; 2 East Kent Hospitals NHS Trust, Canterbury, UK; 3 Kings College London, London, UK

## Abstract

**Background:**

The Contact Heat Evoked Potential Stimulator (CHEPS) utilises rapidly delivered heat pulses with adjustable peak temperatures to stimulate the differential warm/heat thresholds of receptors expressed by Aδ and C fibres. The resulting evoked potentials can be recorded and measured, providing a useful clinical tool for the study of thermal and nociceptive pathways. Concurrent recording of contact heat evoked potentials using electroencephalogram (EEG) and functional magnetic resonance imaging (fMRI) has not previously been reported with CHEPS. Developing simultaneous EEG and fMRI with CHEPS is highly desirable, as it provides an opportunity to exploit the high temporal resolution of EEG and the high spatial resolution of fMRI to study the reaction of the human brain to thermal and nociceptive stimuli.

**Methods:**

In this study we have recorded evoked potentials stimulated by 51°C contact heat pulses from CHEPS using EEG, under normal conditions (baseline), and during continuous and simultaneous acquisition of fMRI images in ten healthy volunteers, during two sessions. The pain evoked by CHEPS was recorded on a Visual Analogue Scale (VAS).

**Results:**

Analysis of EEG data revealed that the latencies and amplitudes of evoked potentials recorded during continuous fMRI did not differ significantly from baseline recordings. fMRI results were consistent with previous thermal pain studies, and showed Blood Oxygen Level Dependent (BOLD) changes in the insula, post-central gyrus, supplementary motor area (SMA), middle cingulate cortex and pre-central gyrus. There was a significant positive correlation between the evoked potential amplitude (EEG) and the psychophysical perception of pain on the VAS.

**Conclusion:**

The results of this study demonstrate the feasibility of recording contact heat evoked potentials with EEG during continuous and simultaneous fMRI. The combined use of the two methods can lead to identification of distinct patterns of brain activity indicative of pain and pro-nociceptive sensitisation in healthy subjects and chronic pain patients. Further studies are required for the technique to progress as a useful tool in clinical trials of novel analgesics.

## Background

Functional magnetic resonance imaging (fMRI) has developed into a tool that is extensively used in non-invasive brain imaging. It provides information about cerebro-vascular activity throughout the whole brain with excellent spatial localisation, yet it is limited by the poor temporal resolution it offers, which is in the order of seconds. On the other hand, electroencephalogram (EEG) is recorded directly from the scalp of the subject and can provide information about neurophysiological activity with a very precise temporal resolution, in the order of milliseconds. The disadvantage of EEG however, is that localisation of the source of electrical activity within the brain is quite difficult.

Therefore the integration of these two techniques is highly desirable, and would allow the exploitation of the advantages of both techniques – high spatial resolution of fMRI and high temporal resolution of EEG. The technique would be widely applicable in all areas of neurophysiological research, but particularly in pain studies, where combined use of the two techniques could lead to the identification of distinct patterns of brain activity indicative of pain and pro-nociceptive sensitisation in healthy subjects and chronic pain patients. These patterns could prove useful in the assessment of the analgesic efficacy of novel analgesic compounds, adding to the desirability of co-registration of pain evoked potentials (such as those stimulated by contact heat) with EEG and fMRI.

Objective markers of pain could be less variable and/or more sensitive to analgesic treatments (i.e. EPs, fMRI). The present study enables the use of these markers to simultaneously assess pharmacodynamic-pharmacokinetic relationships and antihyperalgesic activity in single dose studies in experimental pain models in humans, and in pain patients (e.g. of novel agents that block TRPV1, the heat and capsaicin receptor). Pain biomarkers are needed to provide early pivotal information on efficacy, dose-response and time-course of TRPV1 antagonists, for strategic rapid and cost-effective drug development. Further, neuropathic pain patients would be expected to have reduced pain-evoked potential amplitudes but increased fMRI activation, different from volunteer or inflammatory models/conditions.

The use of simultaneous EEG and fMRI in pharmacological studies of novel analgesics would allow (i) measurement of the combined response to the same pharmacological intervention when the plasma concentration of the analgesic is at its peak (i.e. at the same time-course); (ii) recordings can be analysed at the single subject level, reducing inter-session variability, which is well recognised with regard to pain scores, and (iii) selection of the parameter which enables possible reduction in dose in clinical trials.

Simultaneous EEG and fMRI has already been utilised in studies of epilepsy [1], sleep [2], studies of human alpha activity [3,4] and the study of auditory [5,6], visual [7,8], and motor activity [9]. In the field of pain research, laser and electrically stimulated pain evoked potentials have been successfully co-registered using EEG and fMRI [10,11], but CHEPS has the advantages of a larger area of stimulation, the ability to apply repetitive stimuli to the same cutaneous area without inducing erythema, and simplicity of use in the clinic (i.e. no eye protection required, easy to move location); however, one disadvantage of CHEPS is that in order to avoid habituation the thermode must be physically moved between stimuli.

Simultaneous EEG/fMRI is a readily feasible application yet the MR environment poses a number of technical challenges, limitations and safety issues (for a review of technical and safety issues see [12,13]). The gradient switching fields and radiofrequency (RF) pulses of the MR scanner can create currents in conducting loops that can potentially cause heating of the electrodes and burns at the point of contact with the scalp of the subject. Therefore it is recommended to avoid loops and crossing over of electrode wires, and introduce current limiting resistors to electrode wires.

The gradient fields and RF pulses of the MR scanner can lead to artefacts that can obscure the signal recorded from the EEG, as can movements within the static magnetic field; movement of the head, talking, swallowing and even the pulsatile motion of the heart (ballistocardiogram artefact). The magnet's helium cold pump and gradient coils also produce mechanical vibrations that can be picked up by the EEG wires in the static magnetic field and converted to electrical noise. All these issues need to be addressed and overcome during experimental set-up and processing of EEG and fMRI data, in terms of patient safety and also preserving the quality of the data collected.

Until recently, quantifiable contact heat evoked potentials in EEG had been hard to elicit, due to technical limitations i.e. slow temperature rise and fall times. However, the contact heat evoked potential stimulator (CHEPS) has been designed with a maximum (adjustable) temperature rise time of 70°C/s. CHEPS can stimulate the differential thermal thresholds of receptors innervated by Aδ and C nociceptive nerve fibres, and has been shown to selectively excite these fibre subtypes in human hairy and glabrous skin [14]. The latencies and amplitudes of heat evoked Aδ potentials stimulated by CHEPS have been shown to be robust and reproducible [15,16], despite the disadvantage posed by the slow temperature rise time of the heat stimuli produced by CHEPS (70°C/s compared to a rise time of greater than 1000°C/s reported with lasers) which may lead to a reduction in temporal summation and thus a less synchronous afferent volley [17].

The compatibility of CHEPS with fMRI further widens its scope of application both in healthy volunteers for research purposes and in chronic pain patients. Reproducibility of blood oxygen level dependent (BOLD) responses and pain scores to CHEPS stimulation have already been illustrated in healthy volunteers [18].

In this human volunteer study we have assessed the feasibility of monitoring contact heat evoked Aδ potentials with simultaneous EEG and fMRI, and determined their relationship to the ratings of evoked pain.

## Methods

### Subjects

Ten healthy volunteers were recruited to take part in this feasibility study (6 female, 4 male). The average age of participants was 27.7 years (range 22 – 35 years). Informed consent was taken from all subjects prior to commencement of the study and the study itself was approved by Hammersmith and Queen Charlotte's & Chelsea Research Ethics Committee (REC reference 06/Q0404/9).

### Heat stimuli

Contact heat stimulation was performed using CHEPS (Medoc Ltd, Ramat Yishai, Israel) with a thermode area of 572.5 mm^2^, and a heating thermo-foil (Minco Products, Inc., Minneapolis, MN) covered with a 25 μm layer of thermo conductive plastic (Kapton^®^, thermal conductivity at 23°C of 0.1 – 0.35 W/m/K). The thermode heating rate was up to 70°C/s, the cooling rate up to 40°C/s and the baseline temperature was 32°C.

### Experimental setup

#### Baseline protocol

Baseline recording of evoked potentials was undertaken with the subject lying on the scanner table (outside the magnet) without any scanning taking place. Ten 51°C stimuli of approximately 800 ms duration (approximately 271 ms to reach peak temperature of 51°C and approximately 475 ms to return to baseline [total 746 ms]) and 7 s inter-stimulus interval were applied to the left volar forearm of the subject, and the thermode moved after each stimulus to avoid habituation.

#### fMRI protocol

**Figure 1:**
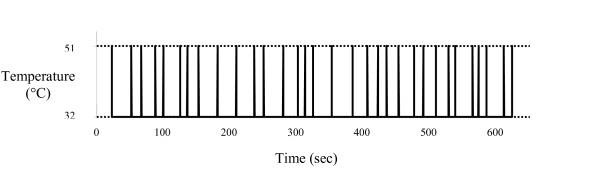
**Event related paradigm**. The protocol during continuous fMRI acquisition consisted of thirty-two 51°C stimuli (from a baseline of 32°C) of approximately 800 ms duration. The inter-stimulus interval varied between 8 and 32 s.

An event-related protocol was used during continuous fMRI acquisition. The protocol consisted of thirty-two 51°C stimuli of approximately 800 ms duration, where the inter-stimulus interval varied between 8 and 32 s. Stimuli were again applied to the left volar forearm, and the thermode moved after each stimulus (Figure 1). A well recognized adverse feature of CHEPS is that placement of the thermode on the skin can cause concomitant activation of mechanoreceptors in the skin. Movement of the thermode after each heat pulse was deemed necessary in order to prevent habituation of the subjects to the stimulus [19], however as this is not synchronised with the paradigm we do not anticipate that it will result in any BOLD response.

Baseline and fMRI protocols were repeated on two occasions on all subjects to assess reproducibility.

### Pain ratings

After completion of baseline and fMRI protocols, subjects were asked to rate the pain of contact heat stimulation by CHEPS on a visual analogue scale (VAS), by placing a mark on a line 10 cm in length that was graded from 0 to 10, 0 being not painful at all, and 10 being the worst pain imaginable. The distance of the mark from 0 (in cm) was measured and noted as the pain score.

### Electroencephalogram (EEG) recording

EEG was acquired from a 32 electrode cap using MRI compatible equipment (BrainCap MR, BrainAmp MR Plus 32 Amplifier, BrainProducts GmbH, Munich, Germany) with the subject relaxed and eyes opened. Electrodes were contacted with Abralyt 2000 electrode gel and impedance maintained below 5 k Ω (when the value of the 5 k Ω current limiting safety resistors [in place as standard on each electrode lead, close to the electrode itself] was subtracted). The data was acquired in a mono-polar fashion, which avoids the disadvantages of average reference recordings and allows for re-montaging of data after acquisition. The reference electrode was located in the FCz position, and the ground electrode in the Iz position. Vertical electro-oculogram (EOG) and electro-cardiogram (ECG) were monitored to allow exclusion of traces contaminated by eye blinks and to enable pulse artefact subtraction. EEG signals were digitised and transmitted to acquisition equipment outside the scanner room by optical fibre. For the baseline recording (outside the scanner) a sampling rate of 250 Hz was applied, and for fMRI recording (inside the scanner) a sampling rate of 5000 Hz was applied. Online low and high pass filters (10 s and 250 Hz respectively) were also applied to the EEG data recorded during baseline and fMRI protocols. CHEPS stimulation and scan events were registered on a trigger channel connected to the acquisition equipment.

EEG data was analysed using a dedicated software package (BrainVision Analyser Version 1.05.0002, BrainProducts GmbH, Munich, Germany). The baseline EEG data was filtered (low pass 0.5305 Hz, high pass 40 Hz), segmented around the trigger input from CHEPS, corrected for blinks using the Gratton and Coles correction algorithm, averaged (10 segments), and re-referenced to an average reference. A baseline correction was applied from -200 to 0 ms pre-stimulus.

The EEG data recorded during fMRI was corrected for scan artefacts using an MRI correction solution provided with the analysis software, using subtraction algorithms based on methods originally developed by Allen and colleagues [20]. Scan start points were detected and marked (using average gradient and criterion for continuous scans) and corrected (using template drift correction). Pulse artefacts were also removed using a solution provided with the analysis software (correction by R peaks – demeaned amplitude algorithm used for R peak detection). Independent component analysis (ICA) was carried out on all data sets, and trials contaminated by subject movement or ocular artefacts were identified visually and removed in the ICA back transform. The data was segmented, averaged (33 segments), re-referenced and a baseline correction applied (as above for the baseline recorded data).

All EEG data is reported from the Cz electrode, with an average reference. The latency of heat evoked Aδ potentials was measured from the first definitive negative peak (N2) and the amplitude measured peak to peak – N2 to P2 (the N2 to P2 component was defined as the peak within a time window of 250 – 550 ms).

Graphs were created and statistical tests performed on EEG data using GraphPad Prism (Version 3.02 for Windows, GraphPad Software, San Diego California USA).

### fMRI acquisition

In each scanning session, 275 fMRI scans were acquired on a 1.5 T Siemens Avanto magnetic resonance scanner (Siemens Medical Systems, Erlangen, Germany) with a standard head array coil. 19 slices parallel to the anterior commisure and the posterior commisure (AC/PC line) were acquired using a gradient echo EPI (Echo Planar Imaging) sequence (Repetition time/Echo time (TR/TE) = 2.3 s/53 ms, flip angle = 90°, field-of-view (FOV) = 23 cm, matrix = 64 × 64, voxel size = 3.6 mm × 3.6 mm × 5 mm). A high resolution T1-MP-RAGE anatomical scan was also obtained (TR/TE = 11 ms/5.2 ms, flip angle = 15°, FOV = 23 cm, matrix = 256 × 256, voxel size = 0.9 mm × 0.9 mm × 1 mm).

To achieve synchronization, the trigger output of the scanner was used to initialize the fMRI paradigm and triggers from CHEPS stimulation and the scanner were recorded together with the EEG signals.

### fMRI processing

Processing of fMRI images was performed using SPM5 (Functional Imaging Laboratory, London, UK). Due to T1 saturation effects, the first 5 volumes of each acquisition were discarded, leaving 270 volumes. Each dataset was realigned to correct for any motion during the acquisition, time corrected due to differences in image acquisition time between slices, followed by normalisation to transform the data to match the SPM template. The template images supplied with SPM conform to the space defined by the ICBM, NIH P-20 project, and approximate that of the space described in the atlas of [21]. Finally, images were smoothed using an isotropic Gaussian Kernel (8 mm full-width-at-half-maximum).

Statistical analysis was based on the General Linear Model (GLM). For within subject analysis the scanning paradigm was specified in SPM and a first level model estimation was performed. The event responses were modelled onto a design matrix by specifying their onset times and their duration. For the group analysis of the ten subjects, a canonical, random effects model (RFX) was used to make a population inference (corrected p < 0.05). Spatial extend thresholding (voxel threshold = 135 mm^3^) was carried out to exclude isolated voxels or small groups and only show clusters of activation. An average brain, representative of all 10 subjects was determined by averaging the normalised structural brain volumes. Significantly activated regions resulting from the group analysis were superimposed on the average brain. The location of the activated regions was assessed using SPM Anatomy toolbox.

To assess correlation between fMRI activation and evoked potential amplitude (or VAS scores), values collected for each subject were entered as covariates using simple regression analysis in SPM5.

## Results

### Contact heat evoked potentials

**Figure 2:**
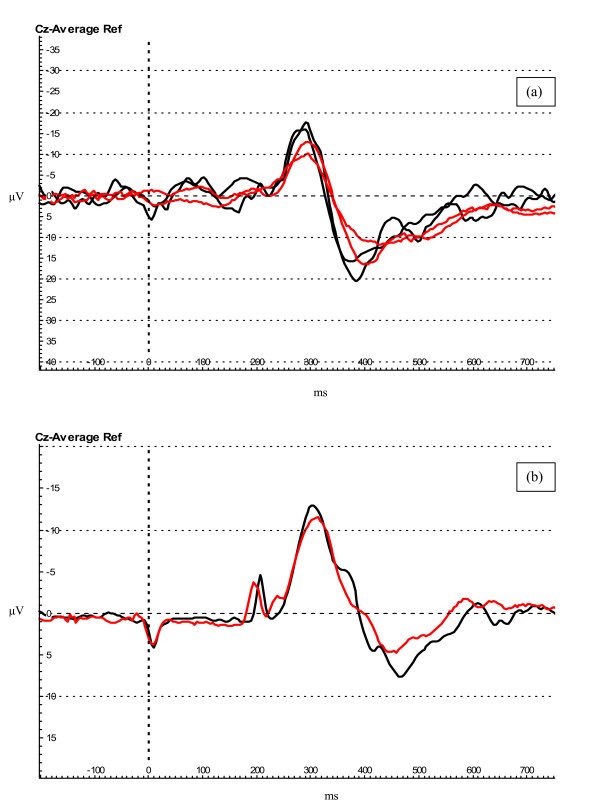
**Evoked potential waveforms**. (a) Evoked potentials recorded during baseline (black traces) and fMRI (red traces) protocols in subject # 5. (b) Evoked potentials (grand average for all subjects) recorded at baseline (1 plus 2) (black trace) and during fMRI (1 plus 2) protocols (red trace). The deflections on the trace occurring at 0 and 250 ms are artefacts produced by the stimulator.

Evoked potentials were successfully recorded using EEG in all subjects during baseline recordings and nine subjects during fMRI acquisition (the data of one subject could not be used due to technical problems). The waveform recorded was similar in both protocols (Figure 2a, b).

The average evoked potential latencies for the first baseline protocol were (mean ± SEM) N2: 0.316 ± 0.009 s, P2: 0.429 ± 0.015 s, and for the second baseline protocol repeated on a separate occasion were N2: 0.322 ± 0.010 s, P2: 0.432 ± 0.013 s. The latencies for the first fMRI protocol were N2: 0.314 ± 0.008 s, P2 0.446 ± 0.017 s, and for the second, N2: 0.317 ± 0.009 s, P2: 0.443 ± 0.015 s. These latencies were approximately 0.100 s longer than those reported in LEP studies, and this is most likely due to the slower temperature rise time of CHEPS (and thus longer stimulus duration) in comparison to laser stimuli, which may lead to slower activation of nociceptors and a reduction of temporal summation [17,22].

**Figure 3:**
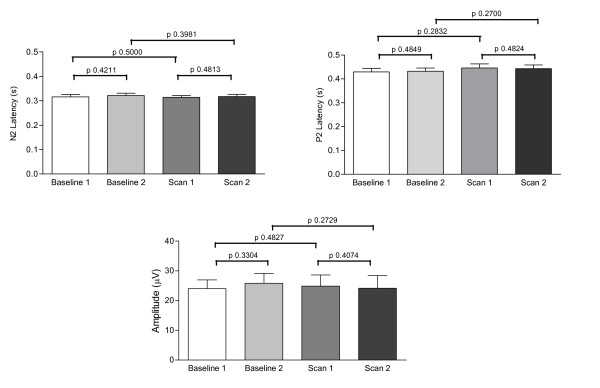
**Latency and amplitude of evoked potentials in baseline and fMRI protocols**. Graphical representation of the N2 and P2 latencies (s) and amplitudes (μV) recorded in the two separate baseline and fMRI sessions. There were no differences in either parameter when recorded under baseline conditions or during continuous fMRI.

The average amplitudes for baseline protocols 1 and 2 were 24.01 ± 2.95 μV and 25.82 ± 3.30 μV respectively. The amplitudes for fMRI protocols were 24.87 ± 3.73 μV for fMRI 1 and 24.14 ± 4.26 μV for fMRI 2. Comparisons of evoked potential latencies and amplitudes revealed no significant differences between the two baseline and fMRI sessions (Figure 3).

Comparisons of pooled baseline and fMRI protocol data did not reveal any difference between latencies and amplitudes (Table 1). There was also no difference in the baseline to peak amplitudes of the baseline and fMRI data (N2 baseline vs. fMRI p = 0.3748; P2 baseline vs. fMRI p = 0.4456).

**Table 1:** Evoked potentials and pain scores recorded during baseline and fMRI protocols in healthy volunteers.

**Protocol**	**N2 latency (s)**	**Amplitude (μV)**	**VAS (0–10)**
Baseline	0.319 ± 0.006	24.87 ± 2.15	5.30 ± 0.53

FMRI	0.317 ± 0.006	24.49 ± 2.77	5.97 ± 0.57

Difference	ns (p = 0.4183)	ns (p = 0.3402)	ns (p = 0.1918)

Mean ± SEM ns = not significant			

Average data (n = 10) for evoked potential latency, amplitude and pain scores during baseline and fMRI protocols. There were no significant differences between the results obtained in the baseline and fMRI sessions.

Differences in overall averages for evoked responses were noted between males and females, with evoked potentials recorded from females having a faster latency (F 0.302 ± 0.004 s vs. M 0.345 ± 0.009 s, p = 0.0003) and a larger amplitude (F 27.62 ± 2.87 μV vs. M 18.47 ± 1.78 μV, p = 0.0286).

No significant correlation between subject age and overall evoked potential latency or amplitude was observed (r_s _= 0.2298, p = 0.2567; r_s _= 0.1043, p = 0.3925 respectively).

### fMRI

**Figure 4:**
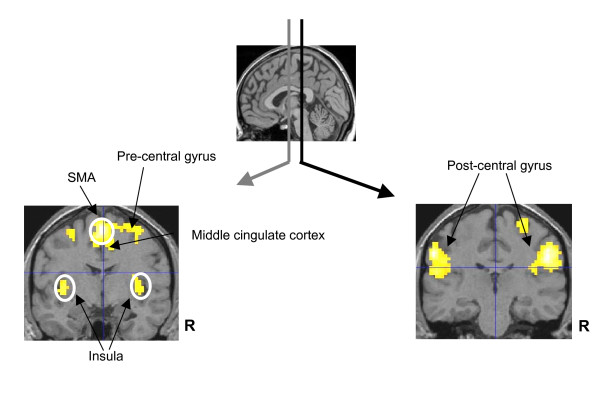
**BOLD fMRI activation during simultaneous EEG**. Noxious heat stimuli at 51°C caused BOLD fMRI responses bilaterally in the insula, post-central gyrus, and SMA and contralateral to the site of stimulation in the middle cingulate cortex and pre-central gyrus. Random effect group analysis results (p < 0.05 corrected) are displayed on structural images in neurological convention.

Group analysis of fMRI data for all ten subjects, collected continuously throughout the fMRI protocol and CHEPS recording revealed areas of Blood Oxygen Level Dependent (BOLD) activation upon CHEPS stimulation bilaterally in the insula, post-central gyrus, and SMA, and contralateral to the site of stimulation (i.e. right) in the middle cingulate cortex and pre-central gyrus (Figure 4, Table 2).

**Table 2:** Areas of BOLD fMRI activation after stimulation using CHEPS.

**Cluster**	**T**	**x**	**y**	**z**	**Anatomical regions**
**Cluster 1 (501 vox)**					

1	9.82	0	-10	65	Left SMA

2	8.66	24	-16	74	Right Superior Frontal Gyrus

3	7.69	30	-25	68	Right Precentral Gyrus

4	7.46	15	-13	68	Right SMA

5	7.43	12	-13	53	Right Middle Cingulate Cortex

**Cluster 2 (366 vox)**					

1	9.55	54	-28	38	Right SupraMarginal Gyrus

2	9.30	57	-25	44	Right Postcentral Gyrus

3	6.50	45	-25	17	Right Heschls Gyrus

**Cluster 3 (357 vox)**					

1	9.58	-51	-37	32	Left SupraMarginal Gyrus

4	8.67	-60	-22	29	Left Postcentral Gyrus

**Cluster 4 (221 vox)**					

1	8.4	-39	-4	5	Left Insula Lobe

2	8.44	-48	-4	17	Left Rolandic Operculum

3	7.15	-51	-1	32	Left Precentral Gyrus

4	5.81	-60	2	11	Left Inferior Frontal Gyrus

**Cluster 5 (208 vox)**					

1	9.27	24	-40	65	Right Postcentral Gyrus

2	9.26	30	-46	56	Right Inferior Parietal Lobule

**Cluster 6 (7 vox)**					

1	6.40	45	11	5	Right Insula Lobe

A summary of activation sites from SPM group analysis.

**Figure 5:**
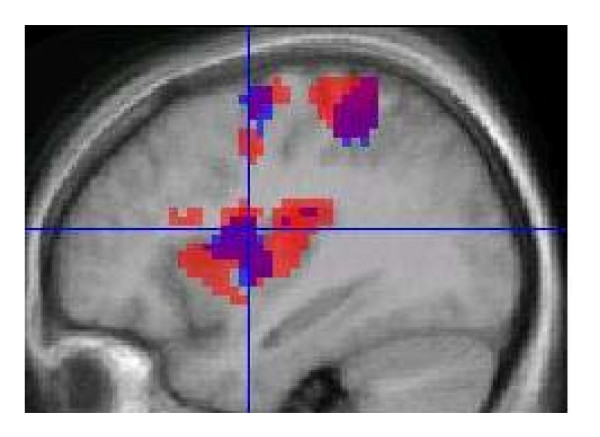
**Reproducibility of fMRI activation over two sessions**. Group results from each fMRI session are superimposed on the average high resolution structural scan. Session 1 is shown in RED, session 2 in BLUE and areas that overlap in PURPLE. There is an overlap in the insula, post-central and pre-central gyrus.

FMRI responses to CHEPS heat stimulation are reproducible across multiple sessions and this is demonstrated in Figure 5 showing group results of each session

Although the thermode was moved randomly, it is possible that there could be some contribution of the touch sensation to the measured BOLD response. To avoid this, the movement of the thermode should be modelled as a nuisance covariate in future experiments.

### Pain ratings

Stimulation at 51°C with the CHEPS probe elicited a painful sensation in all ten subjects. There was no significant difference between reported VAS ratings for baseline and fMRI protocols (Table 1).

Female subjects were noted to have a significantly higher VAS than males for CHEPS stimulation (p = 0.0416). No correlation between age and average reported VAS was observed (r_s _= 0.1534, p = 0.3410).

### Correlations

**Figure 6:**
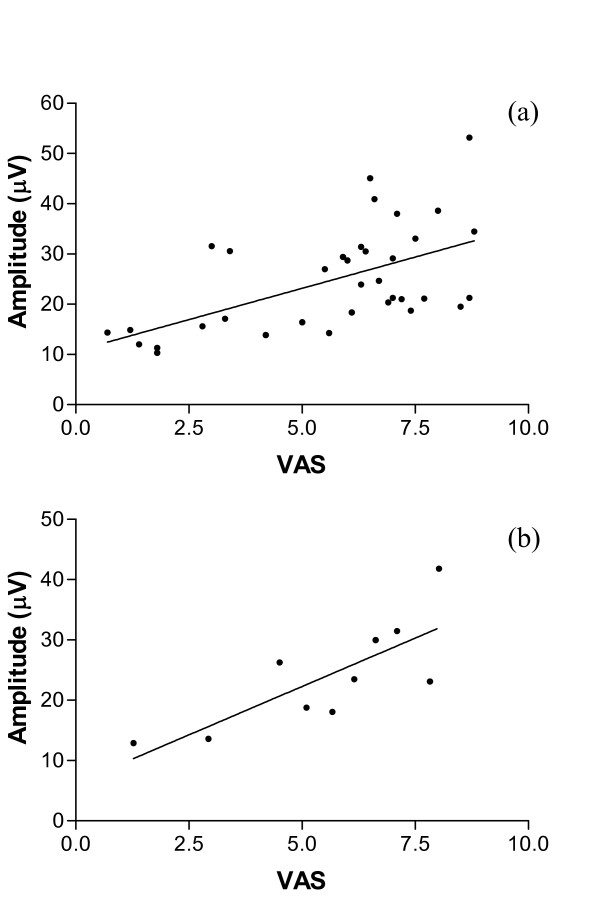
**Correlation between evoked potential amplitude and pain score**. (a) Correlation of amplitude and pain score data for all baseline and fMRI sessions (n = 40) revealed a significant positive relationship (r_s _= 0.5956, p < 0.0001). (b) A significant, positive correlation (r_s _= 0.7697, p = 0.0063) was also seen when the baseline and fMRI data was pooled to give an overall evoked potential amplitude and VAS score for each subject (n = 10).

Correlation of evoked potential amplitude with VAS (for all data, baseline and fMRI protocols repeated twice) revealed a significant, positive relationship, r_s _= 0.5956, p < 0.0001 (Figure 6a). A significant, positive correlation was also seen when the baseline and fMRI data was pooled to give an overall evoked potential amplitude and VAS score for each of the ten subjects, r_s _= 0.7697, p = 0.0063 (Figure 6b).

The regression analysis in SPM showed no correlation between BOLD signal and evoked potential amplitudes or VAS scores.

## Discussion

In this study we have demonstrated that it is feasible to record brain activity in response to noxious thermal stimulation (51°C contact heat) with CHEPS, using simultaneous EEG and fMRI. The EEG recorded during fMRI acquisition was unaffected by the additional processing steps required to remove MRI related artefacts. These artefacts can contaminate the raw data and their removal can compromise the quality of the EEG signal. However, our simultaneously acquired EEG revealed heat evoked Aδ potentials that were reproducible across the two separate fMRI sessions. Although the aim of this feasibility study was not to directly compare the measurements inside and outside the scanner, the amplitudes and latencies recorded inside the scanner were consistent with those recorded in two baseline (control) sessions (Figure 3, Table 1), suggesting there was no signal degradation or reduction of signal to noise ratio caused by conditions in the MRI environment or additional processing requirements. This is in agreement with a similar study using laser-evoked pain conducted by Iannetti and colleagues in 2005, which also showed no variation in amplitude or latency of Aδ evoked potentials inside and outside the scanner. A study of auditory evoked potentials with concurrent fMRI has shown a difference in the latency and amplitude of the N1 component of the event related potential, with longer latencies and higher amplitudes recorded outside the scanner, however no difference in latency or amplitude of the P300 component of the response [5].

To avoid MRI induced artefacts contaminating EEG data, some groups have recorded EEG and fMRI separately [23], or employed an "interleaved" approach, where EEG is recorded during gaps in fMRI image acquisition to avoid artefacts in the EEG data, or short "burst" or "sparse" fMRI protocols are used, avoiding EEG data collection during image acquisition [11,20,24-28]. Despite avoiding most of the technical issues of using truly simultaneous EEG and fMRI, these interleaved protocols have theoretical drawbacks – the two methods are not monitoring the same neurophysiological event when they are recorded separately from one another, which is a disadvantage when the brain activity being studied is in any way unpredictable (such as spike activity in epilepsy, or sleep studies) or changeable (effects of habituation or learning).

Our contact heat evoked potential data has also highlighted a possible gender related difference in the latencies and amplitudes of responses – with female volunteers exhibiting an earlier latency and larger amplitude of response than male volunteers, and also a higher reported VAS score after CHEPS stimulation, an effect that has previously been noted [29]. However, in contrast to this study (which was also conducted in volunteers), there is no effect of subject age upon the latency and amplitude of evoked responses in our data set, or the VAS reported after contact heat stimulation. This may be due to the limited age range in our study, or the small number of subjects recruited. If the number of subjects was increased and the age range broadened, then an age dependent effect may be observed.

Prior to scanning volunteers we used phantom QA tests to assess whether the EEG recording equipment and the CHEPS stimulation device affected the quality of the fMRI images. This included signal-to-noise, ghosting measurements and visual inspection of any artefacts. Our results showed that the fMRI images acquired alongside evoked potential recordings were unaffected by the simultaneous EEG acquisition, and were not impaired by the presence of the EEG recording and CHEPS equipment inside the scanner.

The results we obtained agree with previously published data showing patterns of brain activation after nociceptive and noxious thermal stimulation [10,11,18,30-34], with BOLD changes apparent in the insula, post-central gyrus, SMA, middle cingulate cortex and pre-central gyrus (Figure 4). Cooling of the CHEPS thermode back to baseline temperature will activate cool sensitive-fibres and this could potentially affect the BOLD response; however, the fMRI changes we observed were similar to those obtained with other methods of stimulation with noxious stimuli (these would be primarily related to the initial upstroke of the heat stimulus).

In accord with other studies of pain-evoked potentials, our data has revealed a significant positive correlation between evoked potential amplitude and reported pain scores or pain intensity [15,29,32,35,36] (Figure 6a, b). However, this simple positive relationship with subjective pain scores was not present in the fMRI signal change data, even when regions of interest were analysed separately. This is unlike a recent study of painful electrical stimulation, which showed a positive correlation between stimulus correlated BOLD responses in a network of cortical and cerebellar areas and reported pain intensity [11], and other studies using fMRI that have shown signal changes to correlate with pain intensity, or subjective experience of pain [37,38]. However these studies have either grouped subjects according to their sensitivity to heat stimuli [38], set stimuli according to individual thresholds [11], or used various stimulus intensities [37]. Our fMRI protocol used a stimulus at only one intensity for all subjects in the study, which may explain why no correlation with pain scores could be seen in our fMRI data. In protocols that lead on from this feasibility study stimuli at or just above the pain threshold for each individual will be used. While correlations were made with VAS scores as they are widely used in clinical trials of analgesics, these must be regarded with caution, as VAS scores are ordinal and not linear measures of pain perception – further studies and analyses using an interval scale (Rasch model) would be of interest [39]).

## Conclusion

This study demonstrates the feasibility of recording contact heat evoked potentials with simultaneous EEG and fMRI. Evoked potentials monitored inside the MRI scanner were similar to those recorded under baseline conditions and were highly reproducible on two occasions. We have also demonstrated a linear relationship between evoked potential amplitude and VAS score, as shown in previous volunteer studies. fMRI group analysis showed BOLD activation in areas shown to be associated with nociception in previous publications, but there was no simple correlation of regional fMRI signal changes with individual pain scores – suggesting changes in BOLD signal may reflect later processing in cerebral pathways rather than encoding pain intensity in a linear fashion. Further studies are in progress to demonstrate that simultaneous CHEPS-evoked potentials and fMRI is a useful tool in clinical trials of novel analgesics.

## Competing interests

The authors declare that they have no competing interests.

## Authors' contributions

KR was involved in setting up and carrying out the study, data analysis and writing the manuscript. AP was involved in setting up and carrying out the study, data analysis and writing the manuscript. CG was involved in carrying out the study and data analysis. MT was involved in setting up and carrying out the study and data analysis. DA was involved in setting up the study. RS was involved in carrying out the study. DM helped conceive the study and participated in its design and co-ordination, interpretation, and completion of the manuscript. PA conceived the study and participated in its design and co-ordination, interpretation, and completion of the manuscript. All authors read and approved the manuscript.

## Pre-publication history

The pre-publication history for this paper can be accessed here:


